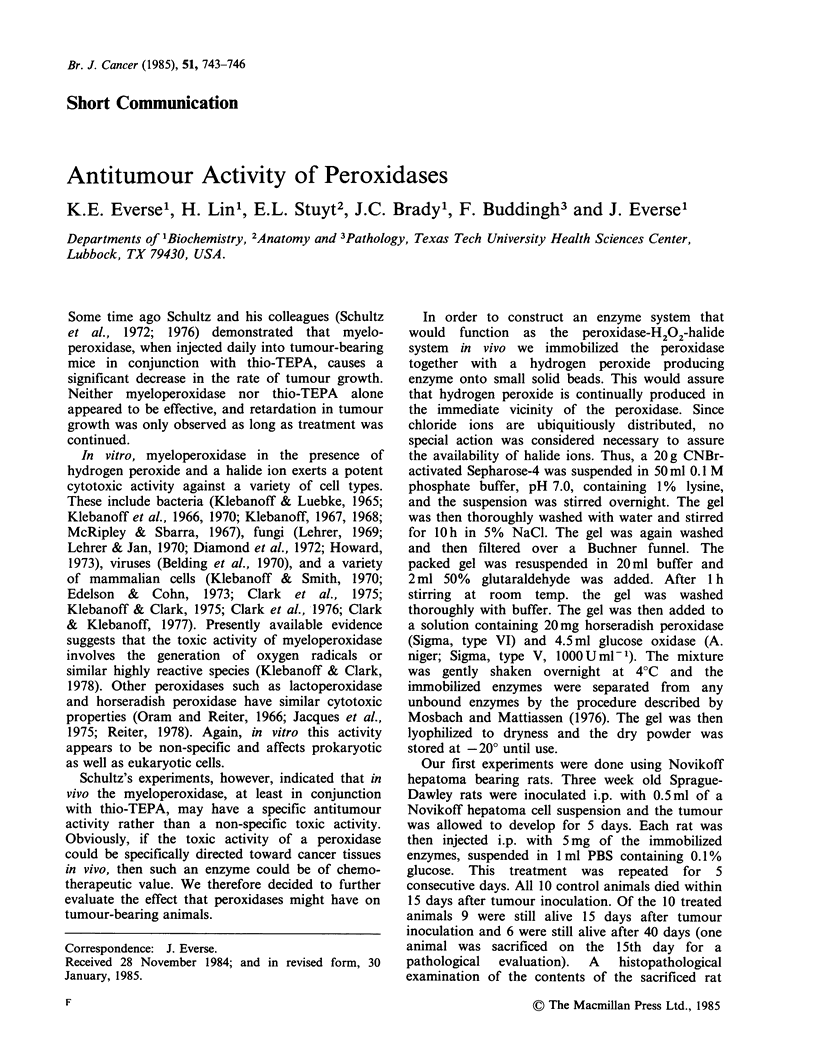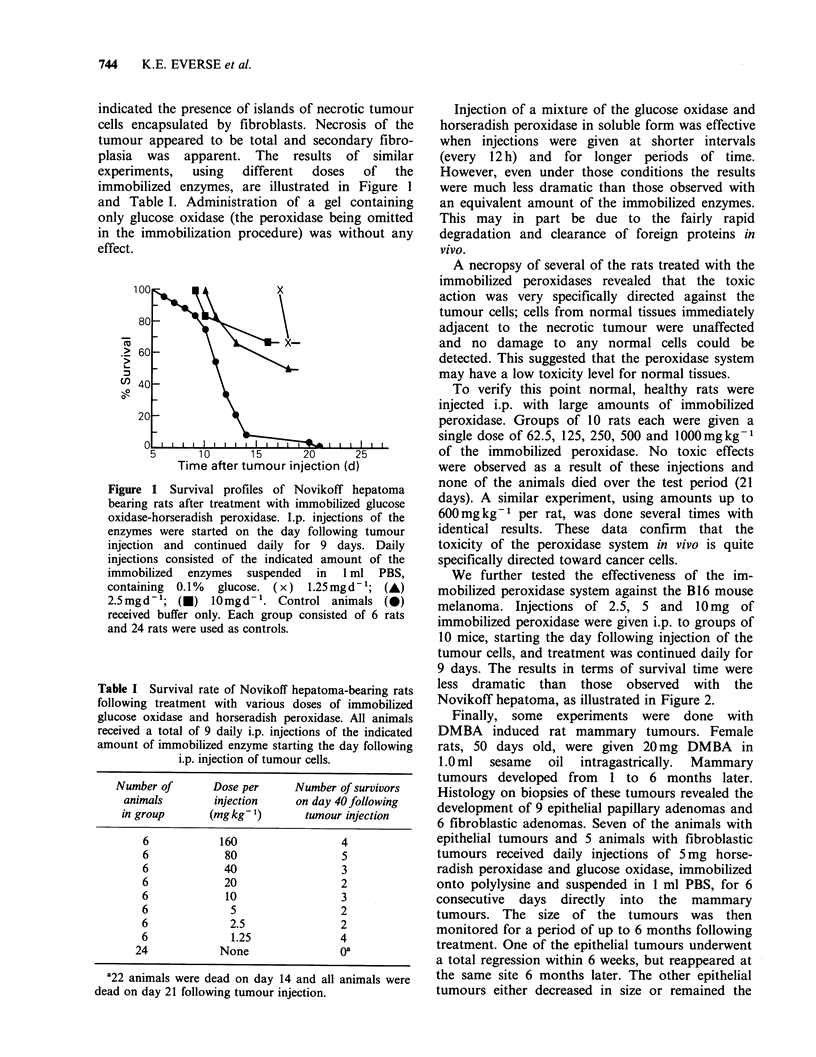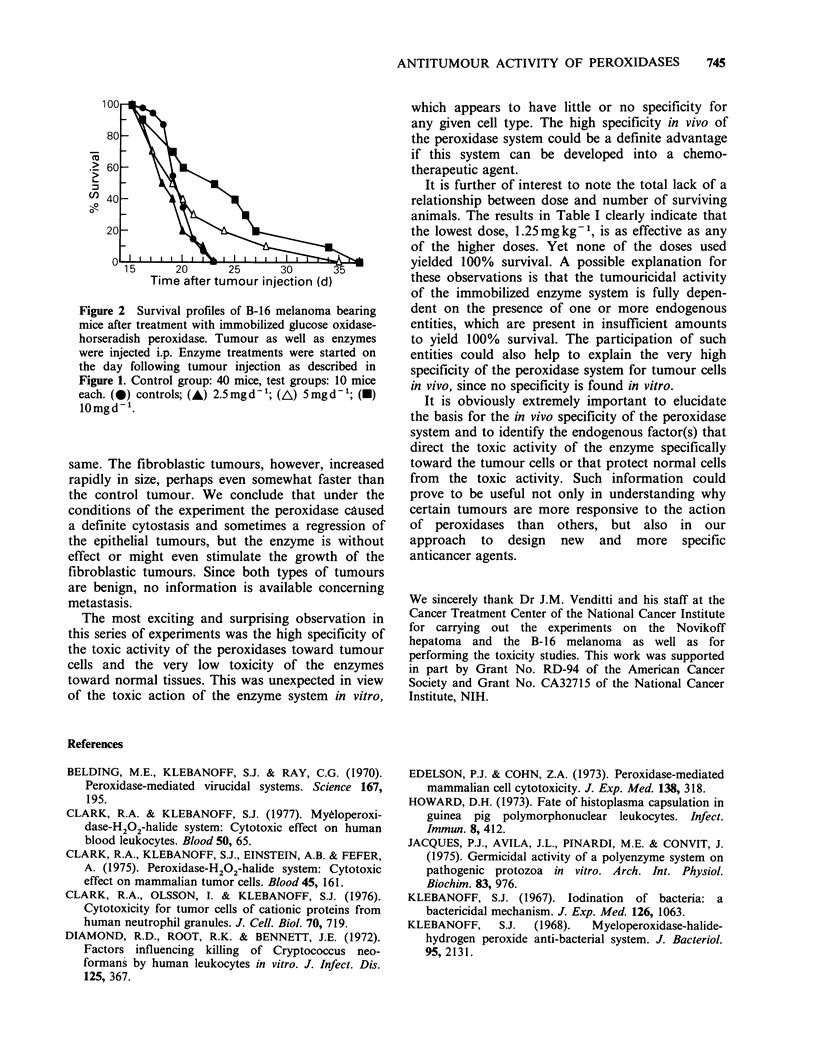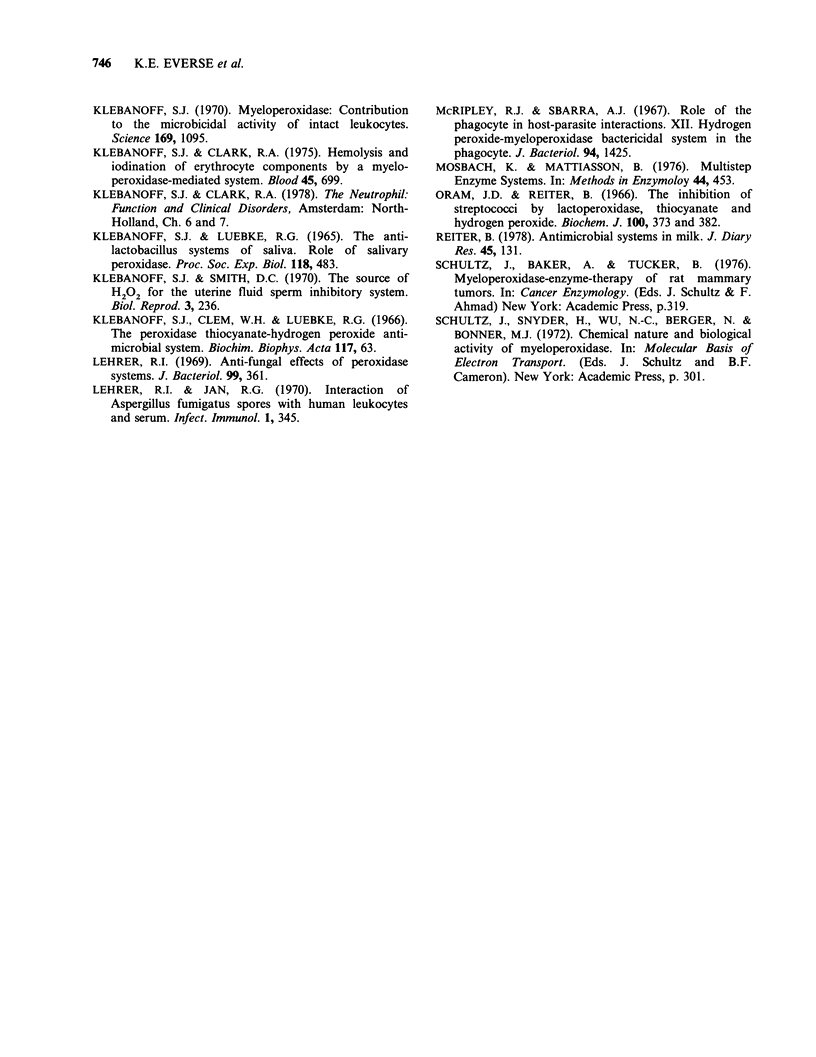# Antitumour activity of peroxidases.

**DOI:** 10.1038/bjc.1985.113

**Published:** 1985-05

**Authors:** K. E. Everse, H. Lin, E. L. Stuyt, J. C. Brady, F. Buddingh, J. Everse


*Br. J. Cancer* (1985), **51**, 743–746

**Short Communication**

### Antitumour Activity of Peroxidases

K.E. Everse[1], H. Lin[1], E.L. Stuyt[2], J.C. Brady[1], F. Buddingh[3] and J. Everse[1]

*Departments of [1]Biochemistry, [2]Anatomy and [3]Pathology, Texas Tech University Health Sciences Center, Lubbock, TX 79430, USA.*

Some time ago Schultz and his colleagues (Schultz *et al.*, 1972; 1976) demonstrated that myeloperoxidase, when injected daily into tumour-bearing mice in conjunction with thio-TEPA, causes a significant decrease in the rate of tumour growth. Neither myeloperoxidase nor thio-TEPA alone appeared to be effective, and retardation in tumour growth was only observed as long as treatment was continued.

*In vitro,* myeloperoxidase in the presence of hydrogen peroxide and a halide ion exerts a potent cytotoxic activity against a variety of cell types. These include bacteria (Klebanoff & Luebke, 1965; Klebanoff *et al.*, 1966, 1970; Klebanoff, 1967, 1968; McRipley & Sbarra, 1967), fungi (Lehrer, 1969; Lehrer & Jan, 1970; Diamond *et al.*, 1972; Howard, 1973), viruses (Belding *et al.*, 1970), and a variety of mammalian cells (Klebanoff & Smith, 1970; Edelson & Cohn, 1973; Clark *et al.*, 1975; Klebanoff & Clark, 1975; Clark *et al.*, 1976; Clark & Klebanoff, 1977). Presently available evidence suggests that the toxic activity of myeloperoxidase involves the generation of oxygen radicals or similar highly reactive species (Klebanoff & Clark, 1978). Other peroxidases such as lactoperoxidase and horseradish peroxidase have similar cytotoxic properties (Oram and Reiter, 1966; Jacques *et al.*, 1975; Reiter, 1978). Again, *in vitro* this activity appears to be non-specific and affects prokaryotic as well as eukaryotic cells.

Schultz's experiments, however, indicated that *in vivo* the myeloperoxidase, at least in conjunction with thio-TEPA, may have a specific antitumour activity rather than a non-specific toxic activity. Obviously, if the toxic activity of a peroxidase could be specifically directed toward cancer tissues *in vivo,* then such an enzyme could be of chemotherapeutic value. We therefore decided to further evaluate the effect that peroxidases might have on tumour-bearing animals.

In order to construct an enzyme system that would function as the peroxidase-$H_2O_2$-halide system *in vivo* we immobilized the peroxidase together with a hydrogen peroxide producing enzyme onto small solid beads. This would assure that hydrogen peroxide is continually produced in the immediate vicinity of the peroxidase. Since chloride ions are ubiquitiously distributed, no special action was considered necessary to assure the availability of halide ions. Thus, a 20 g CNBr-activated Sepharose-4 was suspended in 50 ml 0.1 M phosphate buffer, pH 7.0, containing 1% lysine, and the suspension was stirred overnight. The gel was then thoroughly washed with water and stirred for 10 h in 5% NaCl. The gel was again washed and then filtered over a Buchner funnel. The packed gel was resuspended in 20 ml buffer and 2 ml 50% glutaraldehyde was added. After 1 h stirring at room temp. the gel was washed thoroughly with buffer. The gel was then added to a solution containing 20 mg horseradish peroxidase (Sigma, type VI) and 4.5 ml glucose oxidase (A. niger; Sigma, type V, 1000 U $ml^{-1}$). The mixture was gently shaken overnight at 4°C and the immobilized enzymes were separated from any unbound enzymes by the procedure described by Mosbach and Mattiassen (1976). The gel was then lyophilized to dryness and the dry powder was stored at −20° until use.

Our first experiments were done using Novikoff hepatoma bearing rats. Three week old Sprague-Dawley rats were inoculated i.p. with 0.5 ml of a Novikoff hepatoma cell suspension and the tumour was allowed to develop for 5 days. Each rat was then injected i.p. with 5 mg of the immobilized enzymes, suspended in 1 ml PBS containing 0.1% glucose. This treatment was repeated for 5 consecutive days. All 10 control animals died within 15 days after tumour inoculation. Of the 10 treated animals 9 were still alive 15 days after tumour inoculation and 6 were still alive after 40 days (one animal was sacrificed on the 15th day for a pathological evaluation). A histopathological examination of the contents of the sacrificed rat

Correspondence: J. Everse.
Received 28 November 1984; and in revised form, 30 January, 1985.

© The Macmillan Press Ltd., 1985

indicated the presence of islands of necrotic tumour cells encapsulated by fibroblasts. Necrosis of the tumour appeared to be total and secondary fibroplasia was apparent. The results of similar experiments, using different doses of the immobilized enzymes, are illustrated in Figure 1 and Table I. Administration of a gel containing only glucose oxidase (the peroxidase being omitted in the immobilization procedure) was without any effect.

**Figure 1** Survival profiles of Novikoff hepatoma bearing rats after treatment with immobilized glucose oxidase-horseradish peroxidase. I.p. injections of the enzymes were started on the day following tumour injection and continued daily for 9 days. Daily injections consisted of the indicated amount of the immobilized enzymes suspended in 1 ml PBS, containing 0.1% glucose. (×) $1.25 mg d^{-1}$; (▲) $2.5 mg d^{-1}$; (■) $10 mg d^{-1}$. Control animals (●) received buffer only. Each group consisted of 6 rats and 24 rats were used as controls.

**Table I** Survival rate of Novikoff hepatoma-bearing rats following treatment with various doses of immobilized glucose oxidase and horseradish peroxidase. All animals received a total of 9 daily i.p. injections of the indicated amount of immobilized enzyme starting the day following i.p. injection of tumour cells.

| *Number of animals in group* | *Dose per injection* ($mg kg^{-1}$) | *Number of survivors on day 40 following tumour injection* |
|---|---|---|
| 6 | 160 | 4 |
| 6 | 80 | 5 |
| 6 | 40 | 3 |
| 6 | 20 | 2 |
| 6 | 10 | 3 |
| 6 | 5 | 2 |
| 6 | 2.5 | 2 |
| 6 | 1.25 | 4 |
| 24 | None | 0[a] |

[a]22 animals were dead on day 14 and all animals were dead on day 21 following tumour injection.

Injection of a mixture of the glucose oxidase and horseradish peroxidase in soluble form was effective when injections were given at shorter intervals (every 12 h) and for longer periods of time. However, even under those conditions the results were much less dramatic than those observed with an equivalent amount of the immobilized enzymes. This may in part be due to the fairly rapid degradation and clearance of foreign proteins *in vivo*.

A necropsy of several of the rats treated with the immobilized peroxidases revealed that the toxic action was very specifically directed against the tumour cells; cells from normal tissues immediately adjacent to the necrotic tumour were unaffected and no damage to any normal cells could be detected. This suggested that the peroxidase system may have a low toxicity level for normal tissues.

To verify this point normal, healthy rats were injected i.p. with large amounts of immobilized peroxidase. Groups of 10 rats each were given a single dose of 62.5, 125, 250, 500 and $1000 mg kg^{-1}$ of the immobilized peroxidase. No toxic effects were observed as a result of these injections and none of the animals died over the test period (21 days). A similar experiment, using amounts up to $600 mg kg^{-1}$ per rat, was done several times with identical results. These data confirm that the toxicity of the peroxidase system *in vivo* is quite specifically directed toward cancer cells.

We further tested the effectiveness of the immobilized peroxidase system against the B16 mouse melanoma. Injections of 2.5, 5 and 10 mg of immobilized peroxidase were given i.p. to groups of 10 mice, starting the day following injection of the tumour cells, and treatment was continued daily for 9 days. The results in terms of survival time were less dramatic than those observed with the Novikoff hepatoma, as illustrated in Figure 2.

Finally, some experiments were done with DMBA induced rat mammary tumours. Female rats, 50 days old, were given 20 mg DMBA in 1.0 ml sesame oil intragastrically. Mammary tumours developed from 1 to 6 months later. Histology on biopsies of these tumours revealed the development of 9 epithelial papillary adenomas and 6 fibroblastic adenomas. Seven of the animals with epithelial tumours and 5 animals with fibroblastic tumours received daily injections of 5 mg horseradish peroxidase and glucose oxidase, immobilized onto polylysine and suspended in 1 ml PBS, for 6 consecutive days directly into the mammary tumours. The size of the tumours was then monitored for a period of up to 6 months following treatment. One of the epithelial tumours underwent a total regression within 6 weeks, but reappeared at the same site 6 months later. The other epithelial tumours either decreased in size or remained the

**Figure 2** Survival profiles of B-16 melanoma bearing mice after treatment with immobilized glucose oxidase-horseradish peroxidase. Tumour as well as enzymes were injected i.p. Enzyme treatments were started on the day following tumour injection as described in **Figure 1**. Control group: 40 mice, test groups: 10 mice each. (●) controls; (▲) 2.5 mg $d^{-1}$; (△) 5 mg $d^{-1}$; (■) 10 mg $d^{-1}$.

same. The fibroblastic tumours, however, increased rapidly in size, perhaps even somewhat faster than the control tumour. We conclude that under the conditions of the experiment the peroxidase caused a definite cytostasis and sometimes a regression of the epithelial tumours, but the enzyme is without effect or might even stimulate the growth of the fibroblastic tumours. Since both types of tumours are benign, no information is available concerning metastasis.

The most exciting and surprising observation in this series of experiments was the high specificity of the toxic activity of the peroxidases toward tumour cells and the very low toxicity of the enzymes toward normal tissues. This was unexpected in view of the toxic action of the enzyme system *in vitro*, which appears to have little or no specificity for any given cell type. The high specificity *in vivo* of the peroxidase system could be a definite advantage if this system can be developed into a chemotherapeutic agent.

It is further of interest to note the total lack of a relationship between dose and number of surviving animals. The results in Table I clearly indicate that the lowest dose, 1.25 mg $kg^{-1}$, is as effective as any of the higher doses. Yet none of the doses used yielded 100% survival. A possible explanation for these observations is that the tumouricidal activity of the immobilized enzyme system is fully dependent on the presence of one or more endogenous entities, which are present in insufficient amounts to yield 100% survival. The participation of such entities could also help to explain the very high specificity of the peroxidase system for tumour cells *in vivo*, since no specificity is found *in vitro*.

It is obviously extremely important to elucidate the basis for the *in vivo* specificity of the peroxidase system and to identify the endogenous factor(s) that direct the toxic activity of the enzyme specifically toward the tumour cells or that protect normal cells from the toxic activity. Such information could prove to be useful not only in understanding why certain tumours are more responsive to the action of peroxidases than others, but also in our approach to design new and more specific anticancer agents.

We sincerely thank Dr J.M. Venditti and his staff at the Cancer Treatment Center of the National Cancer Institute for carrying out the experiments on the Novikoff hepatoma and the B-16 melanoma as well as for performing the toxicity studies. This work was supported in part by Grant No. RD-94 of the American Cancer Society and Grant No. CA32715 of the National Cancer Institute, NIH.

### References

BELDING, M.E., KLEBANOFF, S.J. & RAY, C.G. (1970). Peroxidase-mediated virucidal systems. *Science* **167**, 195.

CLARK, R.A. & KLEBANOFF, S.J. (1977). Myèloperoxidase-$H_2O_2$-halide system: Cytotoxic effect on human blood leukocytes. *Blood* **50**, 65.

CLARK, R.A., KLEBANOFF, S.J., EINSTEIN, A.B. & FEFER, A. (1975). Peroxidase-$H_2O_2$-halide system: Cytotoxic effect on mammalian tumor cells. *Blood* **45**, 161.

CLARK, R.A., OLSSON, I. & KLEBANOFF, S.J. (1976). Cytotoxicity for tumor cells of cationic proteins from human neutrophil granules. *J. Cell. Biol.* **70**, 719.

DIAMOND, R.D., ROOT, R.K. & BENNETT, J.E. (1972). Factors influencing killing of Cryptococcus neoformans by human leukocytes *in vitro*. *J. Infect. Dis.* **125**, 367.

EDELSON, P.J. & COHN, Z.A. (1973). Peroxidase-mediated mammalian cell cytotoxicity. *J. Exp. Med.* **138**, 318.

HOWARD, D.H. (1973). Fate of histoplasma capsulation in guinea pig polymorphonuclear leukocytes. *Infect. Immun.* **8**, 412.

JACQUES, P.J., AVILA, J.L., PINARDI, M.E. & CONVIT, J. (1975). Germicidal activity of a polyenzyme system on pathogenic protozoa *in vitro*. *Arch. Int. Physiol. Biochim.* **83**, 976.

KLEBANOFF, S.J. (1967). Iodination of bacteria: a bactericidal mechanism. *J. Exp. Med.* **126**, 1063.

KLEBANOFF, S.J. (1968). Myeloperoxidase-halide-hydrogen peroxide anti-bacterial system. *J. Bacteriol.* **95**, 2131.

KLEBANOFF, S.J. (1970). Myeloperoxidase: Contribution to the microbicidal activity of intact leukocytes. *Science* **169**, 1095.

KLEBANOFF, S.J. & CLARK, R.A. (1975). Hemolysis and iodination of erythrocyte components by a myeloperoxidase-mediated system. *Blood* **45**, 699.

KLEBANOFF, S.J. & CLARK, R.A. (1978). *The Neutrophil: Function and Clinical Disorders,* Amsterdam: North-Holland, Ch. 6 and 7.

KLEBANOFF, S.J. & LUEBKE, R.G. (1965). The antilactobacillus systems of saliva. Role of salivary peroxidase. *Proc. Soc. Exp. Biol.* **118,** 483.

KLEBANOFF, S.J. & SMITH, D.C. (1970). The source of $H_2O_2$ for the uterine fluid sperm inhibitory system. *Biol. Reprod.* **3,** 236.

KLEBANOFF, S.J., CLEM, W.H. & LUEBKE, R.G. (1966). The peroxidase thiocyanate-hydrogen peroxide antimicrobial system. *Biochim. Biophys. Acta* **117,** 63.

LEHRER, R.I. (1969). Anti-fungal effects of peroxidase systems. *J. Bacteriol.* **99,** 361.

LEHRER, R.I. & JAN, R.G. (1970). Interaction of Aspergillus fumigatus spores with human leukocytes and serum. *Infect. Immunol.* **1,** 345.

McRIPLEY, R.J. & SBARRA, A.J. (1967). Role of the phagocyte in host-parasite interactions. XII. Hydrogen peroxide-myeloperoxidase bactericidal system in the phagocyte. *J. Bacteriol.* **94,** 1425.

MOSBACH, K. & MATTIASSON, B. (1976). Multistep Enzyme Systems. In: *Methods in Enzymoloy* **44,** 453.

ORAM, J.D. & REITER, B. (1966). The inhibition of streptococci by lactoperoxidase, thiocyanate and hydrogen peroxide. *Biochem. J.* **100,** 373 and 382.

REITER, B. (1978). Antimicrobial systems in milk. *J. Diary Res.* **45,** 131.

SCHULTZ, J., BAKER, A. & TUCKER, B. (1976). Myeloperoxidase-enzyme-therapy of rat mammary tumors. In: *Cancer Enzymology.* (Eds. J. Schultz & F. Ahmad) New York: Academic Press, p.319.

SCHULTZ, J., SNYDER, H., WU, N.-C., BERGER, N. & BONNER, M.J. (1972). Chemical nature and biological activity of myeloperoxidase. In: *Molecular Basis of Electron Transport.* (Eds. J. Schultz and B.F. Cameron). New York: Academic Press, p. 301.